# 3-[(4-Phenyl­piperazin-1-yl)meth­yl]-5-(thio­phen-2-yl)-2,3-di­hydro-1,3,4-oxa­diazole-2-thione

**DOI:** 10.1107/S1600536813009252

**Published:** 2013-04-10

**Authors:** Ali A. El-Emam, Mohamed A. Al-Omar, Abdul-Rahman M. Al-Obaid, Seik Weng Ng, Edward R. T. Tiekink

**Affiliations:** a Department of Pharmaceutical Chemistry, College of Pharmacy, King Saud University, Riyadh 11451, Saudi Arabia; b Department of Chemistry, University of Malaya, 50603 Kuala Lumpur, Malaysia; c Chemistry Department, Faculty of Science, King Abdulaziz University, PO Box 80203 Jeddah, Saudi Arabia

## Abstract

In the title compound, C_17_H_18_N_4_OS_2_, the 2-thienyl ring is disordered over two co-planar, opposite orientations in a 0.684 (2): 0.316 ratio. The 1,3,4-oxa­diazole ring is almost co-planar with the attached 2-thienyl ring [dihedral angles of 5.34 (19) and 4.8 (5)° for the major and minor components, respectively]. The relative disposition of the thione- and ring-S atoms is *anti* for the major orientation of the 2-thienyl residue. Overall, the shape of the mol­ecule approximates the letter V. In the crystal, a three-dimensional architecture is consolidated by a combination of weak C—H⋯S and C—H⋯π contacts.

## Related literature

For background to the biological properties of 1,3,4-oxa­diazole derivatives, see: Al-Omar (2010 ▶). For a related structure, see: El-Emam *et al.* (2012 ▶).
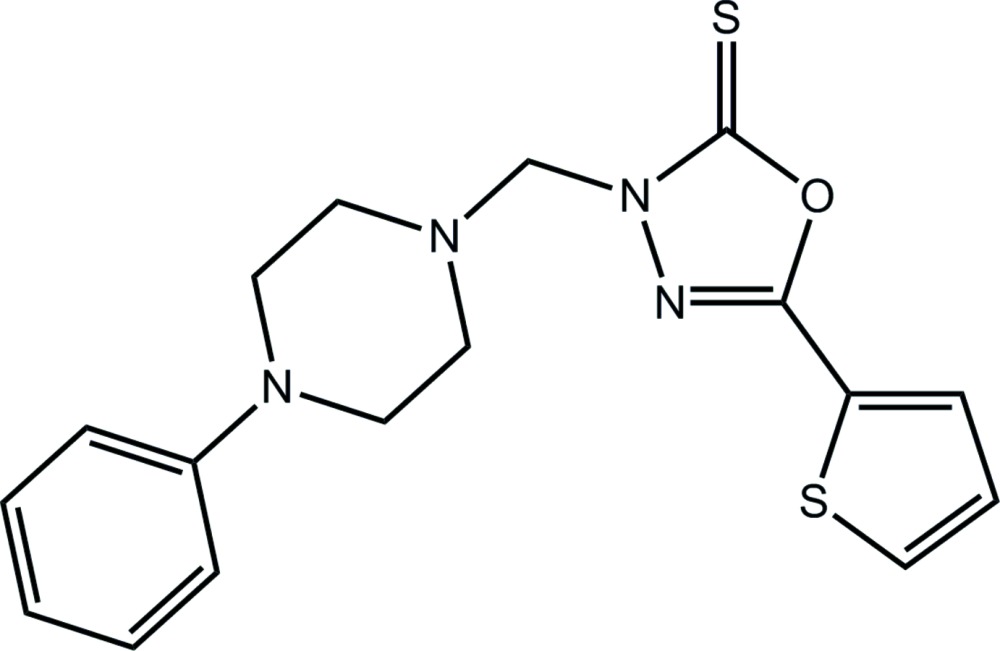


## Experimental

### 

#### Crystal data

C_17_H_18_N_4_OS_2_
*M* *_r_* = 358.47
Monoclinic, 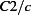

*a* = 26.1721 (17) Å
*b* = 5.7253 (3) Å
*c* = 23.7008 (18) Å
β = 97.802 (6)°
*V* = 3518.5 (4) Å^3^
*Z* = 8
Mo *K*α radiation
μ = 0.31 mm^−1^
*T* = 295 K
0.40 × 0.30 × 0.20 mm

#### Data collection

Agilent SuperNova Dual diffractometer with Atlas detector
Absorption correction: multi-scan (*CrysAlis PRO*; Agilent, 2011 ▶) *T*_min_ = 0.887, *T*_max_ = 1.000
9546 measured reflections
4083 independent reflections
2797 reflections with *I* > 2σ(*I*)
*R*_int_ = 0.025

#### Refinement

*R*[*F* ^2^ > 2σ(*F* ^2^)] = 0.047
*wR*(*F* ^2^) = 0.123
*S* = 1.06
4083 reflections
230 parameters
33 restraints
H-atom parameters constrained
Δρ_max_ = 0.25 e Å^−3^
Δρ_min_ = −0.26 e Å^−3^

### 

Data collection: *CrysAlis PRO* (Agilent, 2011 ▶); cell refinement: *CrysAlis PRO*; data reduction: *CrysAlis PRO*; program(s) used to solve structure: *SHELXS97* (Sheldrick, 2008 ▶); program(s) used to refine structure: *SHELXL97* (Sheldrick, 2008 ▶); molecular graphics: *ORTEP-3 for Windows* (Farrugia, 2012 ▶) and *DIAMOND* (Brandenburg, 2006 ▶); software used to prepare material for publication: *publCIF* (Westrip, 2010 ▶).

## Supplementary Material

Crystal structure: contains datablock(s) global, I. DOI: 10.1107/S1600536813009252/hb7064sup1.cif

Structure factors: contains datablock(s) I. DOI: 10.1107/S1600536813009252/hb7064Isup2.hkl

Supplementary material file. DOI: 10.1107/S1600536813009252/hb7064Isup3.cml

Additional supplementary materials:  crystallographic information; 3D view; checkCIF report

## Figures and Tables

**Table 1:** Hydrogen-bond geometry (Å, °) *Cg*1 is the centroid of the O1,N1,N2,C5,C6 ring

*D*—H⋯*A*	*D*—H	H⋯*A*	*D*⋯*A*	*D*—H⋯*A*
C9—H9*B*⋯S2^i^	0.97	2.77	3.618 (3)	146
C1—H1⋯*Cg*1^ii^	0.93	2.72	3.545 (6)	148
C11—H11*A*⋯*Cg*1^iii^	0.97	2.98	3.600 (2)	123
C15—H15⋯*Cg*2^iii^	0.93	2.95	3.743 (3)	144

Symmetry codes: (i) 
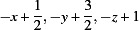
; (ii) 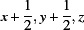; (iii) 
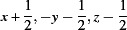
.

**Fig. 1.**
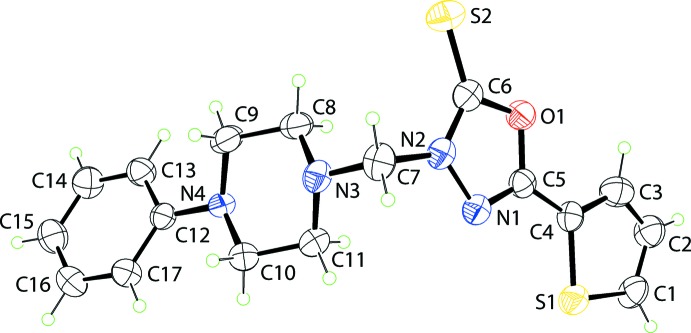
The molecular structure of (I) showing displacement ellipsoids at the 35% probability level. Only the major component of the disordered 2-thienyl ring is shown for reasons of clarity.

**Fig. 2.**
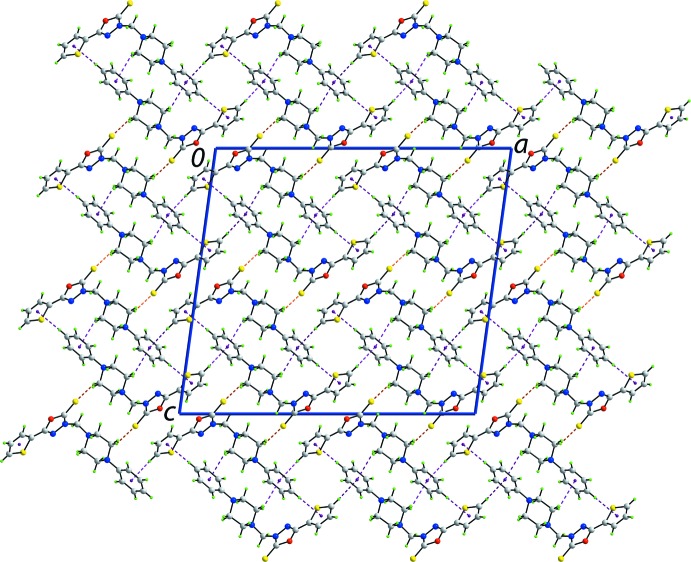
A view of the unit contents in projection down the b axis in (I). The C—H···S and C—H···π contacts are shown as orange and purple dashed lines, respectively.

## supplementary crystallographic information

### Comment

The title compound, (I), was synthesized among a series of 2-thienyl-1,3,4-oxadiazoles and related derivatives as potential anti-microbial agents (Al-Omar, 2010). Herein, the crystal structure of (I) is reported.

In (I), Fig. 1, the 1,3,4-oxadiazole ring is almost planar (r.m.s. deviation = 0.005 Å) and almost co-planar with the attached 2-thienyl ring, forming a dihedral angle of 5.34 (19)° [the equivalent dihedral for the minor component of the disordered 2-thienyl ring is 4.8 (5)°]. The relative disposition of the two S atoms is *anti* for the major component of the 2-thienyl residue. The 4-phenylpiperazinyl residue, in which both *N*-bound substituents occupy equatorial positions in a chair conformation, is linked to the five-membered ring at the methylene-C7 atom so that, overall, the shape of the molecule approximates the letter V. The relative orientations of the 2-thienyl and terminal phenyl rings in (I) are also found in the structure where the 4-phenylpiperazinyl group of (I) is replaced by a *N*-methylanilino residue (El-Emam *et al.*, 2012).

In the crystal, C—H···S interactions combine with phenyl-C—H···π(thienyl), thienyl-C—H···π(phenyl) and piperazinyl-C—H···π(phenyl) contacts to consolidate a three-dimensional architecture, Table 1 and Fig. 2.

### Experimental

1-Phenylpiperazine (324 mg, 2 mmol) and 37% formaldehyde solution (0.5 ml) were added to a solution of 5-(thiophen-2-yl)-1,3,4-oxadiazole-2-thiol (369 mg, 2 mmol) in ethanol (8 ml), and the mixture was stirred at room temperature for 2 h and allowed to stand overnight. The precipitated crude product was filtered, washed with cold ethanol, dried, and crystallized from ethanol to yield 502 mg (70%) of the title compound (I) as crystals. *M*.pt: 404–406 K. Light brown prisms were obtained by slow evaporation of its CHCl_3_:EtOH (1:1; 5 ml) solution at room temperature. ^1^H NMR (CDCl_3_, 500.13 MHz): δ 3.0–3.08 (m, 4H, piperazine-H), 3.18–3.26 (m, 4H, piperazine-H), 5.14 (s, 2H, CH_2_), 6.87–6.96 (m, 3H, Ar—H), 7.18 (t, 1H, thiophene-H, J = 4.3 Hz), 7.22–7.32 (m. 2H, Ar—H), 7.59 (d, 1H, thiophene-H, J = 4.5 Hz), 7.75 (d, 1H, thiophene-H, J = 4.5 Hz). ^13^C NMR (CDCl_3_, 125.76 MHz): δ 49.37, 50.30 (piperazine-C), 70.40 (CH_2_), 116.45, 120.06, 123.67, 128.33, 129.14, 130.77, 130.98, 151.26 (Ar—C & thiophene-C), 155.47 (C═ N), 177.76 (C═S).

### Refinement

The H-atoms were placed in calculated positions [C—H = 0.93 to 0.97 Å, *U*_iso_(H) = 1.2*U*_eq_(C)] and were included in the refinement in the riding model approximation. The thienyl ring is disordered over two positions in a 0.684 (2): 0.316 ratio in respect of four of the five atoms; that connected to the diazoxole ring is ordered. The anisotropic displacement parameters of the primed atoms were set to those of the unprimed ones, and were restrained to be nearly isotropic. Pairs of 1,2-related bond distances were restrained to within 0.01 Å of each other. The thienyl rings were restrained to lie within 0.01 Å of a plane.

### Crystal data

**Table:**

C_17_H_18_N_4_OS_2_	*F*(000) = 1504
*M**_r_* = 358.47	*D*_x_ = 1.353 Mg m^−^^3^
Monoclinic, *C*2/*c*	Mo *K*α radiation, λ = 0.71073 Å
Hall symbol: -C 2yc	Cell parameters from 2605 reflections
*a* = 26.1721 (17) Å	θ = 3.1–27.5°
*b* = 5.7253 (3) Å	µ = 0.31 mm^−^^1^
*c* = 23.7008 (18) Å	*T* = 295 K
β = 97.802 (6)°	Prism, light-brown
*V* = 3518.5 (4) Å^3^	0.40 × 0.30 × 0.20 mm
*Z* = 8	

### Data collection

**Table:**

Agilent SuperNova Dual diffractometer with Atlas detector	4083 independent reflections
Radiation source: SuperNova (Mo) X-ray Source	2797 reflections with *I* > 2σ(*I*)
Mirror monochromator	*R*_int_ = 0.025
Detector resolution: 10.4041 pixels mm^-1^	θ_max_ = 27.6°, θ_min_ = 3.1°
ω scan	*h* = −32→34
Absorption correction: multi-scan (*CrysAlis PRO*; Agilent, 2011)	*k* = −7→5
*T*_min_ = 0.887, *T*_max_ = 1.000	*l* = −16→30
9546 measured reflections	

### Refinement

**Table:**

Refinement on *F*^2^	Primary atom site location: structure-invariant direct methods
Least-squares matrix: full	Secondary atom site location: difference Fourier map
*R*[*F*^2^ > 2σ(*F*^2^)] = 0.047	Hydrogen site location: inferred from neighbouring sites
*wR*(*F*^2^) = 0.123	H-atom parameters constrained
*S* = 1.06	*w* = 1/[σ^2^(*F*_o_^2^) + (0.0496*P*)^2^ + 0.9031*P*] where *P* = (*F*_o_^2^ + 2*F*_c_^2^)/3
4083 reflections	(Δ/σ)_max_ = 0.001
230 parameters	Δρ_max_ = 0.25 e Å^−^^3^
33 restraints	Δρ_min_ = −0.26 e Å^−^^3^

### Special details

**Table:**

Geometry. All e.s.d.'s (except the e.s.d. in the dihedral angle between two l.s. planes) are estimated using the full covariance matrix. The cell e.s.d.'s are taken into account individually in the estimation of e.s.d.'s in distances, angles and torsion angles; correlations between e.s.d.'s in cell parameters are only used when they are defined by crystal symmetry. An approximate (isotropic) treatment of cell e.s.d.'s is used for estimating e.s.d.'s involving l.s. planes.
Refinement. Refinement of *F*^2^ against ALL reflections. The weighted *R*-factor *wR* and goodness of fit *S* are based on *F*^2^, conventional *R*-factors *R* are based on *F*, with *F* set to zero for negative *F*^2^. The threshold expression of *F*^2^ > σ(*F*^2^) is used only for calculating *R*-factors(gt) *etc*. and is not relevant to the choice of reflections for refinement. *R*-factors based on *F*^2^ are statistically about twice as large as those based on *F*, and *R*- factors based on ALL data will be even larger.

### Fractional atomic coordinates and isotropic or equivalent isotropic displacement parameters (Å^2^)

**Table:**

	*x*	*y*	*z*	*U*_iso_*/*U*_eq_	Occ. (<1)
S1	0.51012 (6)	0.4713 (3)	0.35472 (6)	0.0619 (3)	0.684 (2)
S1'	0.51936 (13)	0.8668 (6)	0.43201 (16)	0.0619 (3)	0.316
S2	0.35703 (2)	0.65396 (10)	0.54603 (3)	0.0727 (2)	
O1	0.43151 (5)	0.6708 (2)	0.47998 (5)	0.0534 (3)	
N1	0.41826 (6)	0.3583 (3)	0.42460 (7)	0.0522 (4)	
N2	0.38163 (6)	0.3737 (3)	0.46156 (7)	0.0509 (4)	
N3	0.29660 (6)	0.2250 (3)	0.41864 (7)	0.0556 (4)	
N4	0.22283 (6)	0.3760 (2)	0.32601 (6)	0.0489 (4)	
C1	0.55629 (17)	0.6849 (7)	0.3488 (3)	0.0629 (13)	0.684 (2)
H1	0.5786	0.6799	0.3215	0.076*	0.684 (2)
C2	0.5569 (3)	0.8585 (13)	0.3874 (2)	0.0655 (13)	0.684 (2)
H2	0.5783	0.9889	0.3899	0.079*	0.684 (2)
C3	0.5214 (2)	0.8105 (7)	0.4216 (2)	0.0673 (14)	0.684 (2)
H3	0.5180	0.9064	0.4526	0.081*	0.684 (2)
C1'	0.5583 (6)	0.821 (3)	0.3787 (6)	0.0629 (13)	0.316 (2)
H1'	0.5844	0.9250	0.3726	0.076*	0.316 (2)
C2'	0.5483 (5)	0.627 (2)	0.3469 (7)	0.0655 (13)	0.316 (2)
H2'	0.5649	0.5826	0.3163	0.079*	0.316 (2)
C3'	0.5102 (5)	0.509 (2)	0.3670 (6)	0.0673 (14)	0.316 (2)
H3'	0.4984	0.3649	0.3524	0.081*	0.316 (2)
C4	0.49000 (7)	0.6179 (3)	0.41049 (8)	0.0506 (4)	
C5	0.44667 (7)	0.5396 (3)	0.43703 (8)	0.0481 (4)	
C6	0.38888 (7)	0.5603 (3)	0.49568 (8)	0.0524 (5)	
C7	0.34040 (8)	0.1950 (3)	0.46076 (10)	0.0613 (5)	
H7A	0.3554	0.0431	0.4552	0.074*	
H7B	0.3287	0.1934	0.4979	0.074*	
C8	0.26756 (8)	0.4377 (4)	0.42299 (9)	0.0685 (6)	
H8A	0.2869	0.5698	0.4115	0.082*	
H8B	0.2619	0.4616	0.4622	0.082*	
C9	0.21648 (8)	0.4220 (5)	0.38539 (9)	0.0721 (6)	
H9A	0.1961	0.2980	0.3991	0.086*	
H9B	0.1978	0.5675	0.3876	0.086*	
C10	0.25448 (8)	0.1688 (3)	0.32178 (10)	0.0628 (5)	
H10A	0.2608	0.1490	0.2827	0.075*	
H10B	0.2362	0.0320	0.3325	0.075*	
C11	0.30534 (8)	0.1901 (4)	0.36017 (9)	0.0632 (6)	
H11A	0.3255	0.0493	0.3575	0.076*	
H11B	0.3247	0.3209	0.3480	0.076*	
C12	0.17704 (7)	0.3921 (3)	0.28724 (8)	0.0473 (4)	
C13	0.14489 (8)	0.5860 (3)	0.28800 (9)	0.0573 (5)	
H13	0.1536	0.7033	0.3147	0.069*	
C14	0.10056 (9)	0.6071 (4)	0.25001 (10)	0.0654 (6)	
H14	0.0799	0.7384	0.2515	0.078*	
C15	0.08620 (9)	0.4380 (4)	0.20978 (10)	0.0670 (6)	
H15	0.0560	0.4528	0.1843	0.080*	
C16	0.11754 (9)	0.2471 (4)	0.20823 (9)	0.0658 (6)	
H16	0.1085	0.1314	0.1812	0.079*	
C17	0.16222 (8)	0.2226 (3)	0.24588 (9)	0.0579 (5)	
H17	0.1828	0.0913	0.2437	0.069*	

### Atomic displacement parameters (Å^2^)

**Table:**

	*U*^11^	*U*^22^	*U*^33^	*U*^12^	*U*^13^	*U*^23^
S1	0.0542 (5)	0.0638 (7)	0.0700 (7)	−0.0048 (4)	0.0171 (5)	−0.0069 (5)
S1'	0.0542 (5)	0.0638 (7)	0.0700 (7)	−0.0048 (4)	0.0171 (5)	−0.0069 (5)
S2	0.0815 (4)	0.0786 (4)	0.0626 (4)	0.0100 (3)	0.0265 (3)	0.0018 (3)
O1	0.0542 (8)	0.0540 (7)	0.0521 (8)	−0.0023 (6)	0.0082 (6)	0.0000 (6)
N1	0.0470 (9)	0.0554 (9)	0.0546 (10)	−0.0002 (7)	0.0086 (7)	0.0011 (7)
N2	0.0456 (9)	0.0535 (8)	0.0541 (9)	0.0008 (7)	0.0089 (7)	0.0064 (7)
N3	0.0471 (9)	0.0608 (9)	0.0604 (10)	−0.0044 (8)	0.0124 (8)	0.0048 (8)
N4	0.0457 (8)	0.0541 (8)	0.0491 (9)	−0.0023 (7)	0.0142 (7)	−0.0106 (7)
C1	0.052 (2)	0.058 (3)	0.083 (3)	−0.0088 (18)	0.025 (2)	0.003 (2)
C2	0.061 (2)	0.062 (3)	0.075 (3)	−0.0087 (19)	0.015 (2)	0.0010 (19)
C3	0.078 (3)	0.056 (3)	0.068 (3)	0.006 (2)	0.011 (2)	−0.0046 (19)
C1'	0.052 (2)	0.058 (3)	0.083 (3)	−0.0088 (18)	0.025 (2)	0.003 (2)
C2'	0.061 (2)	0.062 (3)	0.075 (3)	−0.0087 (19)	0.015 (2)	0.0010 (19)
C3'	0.078 (3)	0.056 (3)	0.068 (3)	0.006 (2)	0.011 (2)	−0.0046 (19)
C4	0.0448 (10)	0.0544 (10)	0.0516 (11)	0.0005 (8)	0.0032 (8)	0.0050 (8)
C5	0.0453 (10)	0.0513 (10)	0.0464 (10)	0.0046 (8)	0.0018 (8)	0.0041 (8)
C6	0.0522 (11)	0.0542 (10)	0.0505 (11)	0.0052 (9)	0.0065 (9)	0.0097 (9)
C7	0.0594 (13)	0.0566 (11)	0.0689 (14)	−0.0061 (10)	0.0121 (10)	0.0156 (10)
C8	0.0593 (13)	0.0951 (15)	0.0518 (12)	0.0152 (12)	0.0097 (10)	−0.0165 (11)
C9	0.0537 (12)	0.1097 (17)	0.0543 (13)	0.0173 (12)	0.0127 (10)	−0.0143 (12)
C10	0.0535 (12)	0.0641 (12)	0.0714 (14)	0.0021 (10)	0.0102 (10)	−0.0198 (10)
C11	0.0507 (12)	0.0677 (12)	0.0729 (15)	0.0054 (10)	0.0142 (10)	−0.0162 (10)
C12	0.0469 (10)	0.0488 (9)	0.0491 (10)	−0.0100 (8)	0.0165 (8)	−0.0018 (8)
C13	0.0612 (12)	0.0507 (10)	0.0609 (13)	−0.0047 (10)	0.0120 (10)	−0.0051 (9)
C14	0.0651 (14)	0.0591 (12)	0.0713 (15)	0.0052 (10)	0.0072 (11)	0.0040 (11)
C15	0.0656 (14)	0.0697 (13)	0.0628 (14)	−0.0044 (11)	−0.0026 (11)	0.0052 (11)
C16	0.0708 (14)	0.0665 (13)	0.0575 (13)	−0.0078 (11)	−0.0003 (11)	−0.0100 (10)
C17	0.0606 (12)	0.0551 (10)	0.0585 (12)	−0.0020 (9)	0.0098 (10)	−0.0077 (9)

### Geometric parameters (Å, º)

**Table:**

S1—C4	1.708 (2)	C3'—C4	1.371 (9)
S1—C1	1.738 (3)	C3'—H3'	0.9300
S1'—C4	1.666 (3)	C4—C5	1.441 (3)
S1'—C1'	1.748 (9)	C7—H7A	0.9700
S2—C6	1.636 (2)	C7—H7B	0.9700
O1—C5	1.367 (2)	C8—C9	1.505 (3)
O1—C6	1.377 (2)	C8—H8A	0.9700
N1—C5	1.288 (2)	C8—H8B	0.9700
N1—N2	1.386 (2)	C9—H9A	0.9700
N2—C6	1.338 (2)	C9—H9B	0.9700
N2—C7	1.485 (2)	C10—C11	1.512 (3)
N3—C7	1.425 (3)	C10—H10A	0.9700
N3—C8	1.447 (3)	C10—H10B	0.9700
N3—C11	1.449 (3)	C11—H11A	0.9700
N4—C12	1.411 (2)	C11—H11B	0.9700
N4—C10	1.458 (2)	C12—C13	1.395 (3)
N4—C9	1.463 (2)	C12—C17	1.396 (3)
C1—C2	1.349 (5)	C13—C14	1.374 (3)
C1—H1	0.9300	C13—H13	0.9300
C2—C3	1.342 (7)	C14—C15	1.375 (3)
C2—H2	0.9300	C14—H14	0.9300
C3—C4	1.379 (5)	C15—C16	1.370 (3)
C3—H3	0.9300	C15—H15	0.9300
C1'—C2'	1.349 (8)	C16—C17	1.379 (3)
C1'—H1'	0.9300	C16—H16	0.9300
C2'—C3'	1.344 (10)	C17—H17	0.9300
C2'—H2'	0.9300		
			
C4—S1—C1	90.4 (2)	N2—C7—H7A	108.3
C4—S1'—C1'	86.7 (6)	N3—C7—H7B	108.3
C5—O1—C6	106.05 (14)	N2—C7—H7B	108.3
C5—N1—N2	103.39 (14)	H7A—C7—H7B	107.4
C6—N2—N1	112.28 (15)	N3—C8—C9	109.90 (18)
C6—N2—C7	126.94 (16)	N3—C8—H8A	109.7
N1—N2—C7	120.78 (15)	C9—C8—H8A	109.7
C7—N3—C8	115.60 (17)	N3—C8—H8B	109.7
C7—N3—C11	115.92 (16)	C9—C8—H8B	109.7
C8—N3—C11	109.70 (16)	H8A—C8—H8B	108.2
C12—N4—C10	116.68 (15)	N4—C9—C8	111.87 (16)
C12—N4—C9	114.66 (14)	N4—C9—H9A	109.2
C10—N4—C9	110.64 (17)	C8—C9—H9A	109.2
C2—C1—S1	114.1 (6)	N4—C9—H9B	109.2
C2—C1—H1	122.9	C8—C9—H9B	109.2
S1—C1—H1	122.9	H9A—C9—H9B	107.9
C3—C2—C1	108.2 (7)	N4—C10—C11	110.80 (16)
C3—C2—H2	125.9	N4—C10—H10A	109.5
C1—C2—H2	125.9	C11—C10—H10A	109.5
C2—C3—C4	119.4 (5)	N4—C10—H10B	109.5
C2—C3—H3	120.3	C11—C10—H10B	109.5
C4—C3—H3	120.3	H10A—C10—H10B	108.1
C2'—C1'—S1'	115.9 (14)	N3—C11—C10	110.26 (16)
C2'—C1'—H1'	122.0	N3—C11—H11A	109.6
S1'—C1'—H1'	122.0	C10—C11—H11A	109.6
C3'—C2'—C1'	108.4 (16)	N3—C11—H11B	109.6
C3'—C2'—H2'	125.8	C10—C11—H11B	109.6
C1'—C2'—H2'	125.8	H11A—C11—H11B	108.1
C2'—C3'—C4	114.7 (12)	C13—C12—C17	116.86 (19)
C2'—C3'—H3'	122.7	C13—C12—N4	120.30 (16)
C4—C3'—H3'	122.7	C17—C12—N4	122.82 (17)
C3'—C4—C3	103.2 (6)	C14—C13—C12	121.24 (19)
C3'—C4—C5	126.4 (6)	C14—C13—H13	119.4
C3—C4—C5	130.4 (2)	C12—C13—H13	119.4
C3'—C4—S1'	114.2 (6)	C13—C14—C15	121.3 (2)
C5—C4—S1'	119.33 (17)	C13—C14—H14	119.3
C3—C4—S1	107.6 (2)	C15—C14—H14	119.3
C5—C4—S1	121.91 (15)	C16—C15—C14	118.2 (2)
S1'—C4—S1	118.65 (15)	C16—C15—H15	120.9
N1—C5—O1	113.36 (16)	C14—C15—H15	120.9
N1—C5—C4	127.95 (17)	C15—C16—C17	121.5 (2)
O1—C5—C4	118.66 (16)	C15—C16—H16	119.3
N2—C6—O1	104.91 (15)	C17—C16—H16	119.3
N2—C6—S2	131.15 (15)	C16—C17—C12	120.94 (19)
O1—C6—S2	123.94 (15)	C16—C17—H17	119.5
N3—C7—N2	116.10 (15)	C12—C17—H17	119.5
N3—C7—H7A	108.3		
			
C5—N1—N2—C6	−0.7 (2)	S1'—C4—C5—O1	−1.2 (3)
C5—N1—N2—C7	179.54 (16)	S1—C4—C5—O1	−177.46 (15)
C4—S1—C1—C2	0.5 (3)	N1—N2—C6—O1	0.5 (2)
S1—C1—C2—C3	1.9 (5)	C7—N2—C6—O1	−179.85 (16)
C1—C2—C3—C4	−4.4 (7)	N1—N2—C6—S2	−179.55 (15)
C4—S1'—C1'—C2'	0.4 (9)	C7—N2—C6—S2	0.1 (3)
S1'—C1'—C2'—C3'	−2.3 (13)	C5—O1—C6—N2	−0.01 (18)
C1'—C2'—C3'—C4	3.5 (16)	C5—O1—C6—S2	−179.99 (14)
C2'—C3'—C4—C3	−5.5 (13)	C8—N3—C7—N2	−61.8 (2)
C2'—C3'—C4—C5	175.2 (9)	C11—N3—C7—N2	68.7 (2)
C2'—C3'—C4—S1'	−3.4 (17)	C6—N2—C7—N3	97.8 (2)
C2'—C3'—C4—S1	152 (10)	N1—N2—C7—N3	−82.5 (2)
C2—C3—C4—C3'	6.7 (7)	C7—N3—C8—C9	−166.94 (17)
C2—C3—C4—C5	−174.1 (4)	C11—N3—C8—C9	59.7 (2)
C2—C3—C4—S1'	−164 (2)	C12—N4—C9—C8	−171.40 (17)
C2—C3—C4—S1	4.7 (7)	C10—N4—C9—C8	54.1 (2)
C1'—S1'—C4—C3'	1.7 (11)	N3—C8—C9—N4	−56.9 (3)
C1'—S1'—C4—C3	12 (2)	C12—N4—C10—C11	172.40 (16)
C1'—S1'—C4—C5	−177.1 (6)	C9—N4—C10—C11	−54.1 (2)
C1'—S1'—C4—S1	−0.7 (6)	C7—N3—C11—C10	166.26 (16)
C1—S1—C4—C3'	−26 (9)	C8—N3—C11—C10	−60.6 (2)
C1—S1—C4—C3	−2.6 (4)	N4—C10—C11—N3	57.9 (2)
C1—S1—C4—C5	176.2 (2)	C10—N4—C12—C13	−179.25 (17)
C1—S1—C4—S1'	−0.1 (3)	C9—N4—C12—C13	49.1 (2)
N2—N1—C5—O1	0.74 (19)	C10—N4—C12—C17	−1.0 (2)
N2—N1—C5—C4	−177.59 (17)	C9—N4—C12—C17	−132.6 (2)
C6—O1—C5—N1	−0.5 (2)	C17—C12—C13—C14	0.5 (3)
C6—O1—C5—C4	178.00 (15)	N4—C12—C13—C14	178.91 (17)
C3'—C4—C5—N1	−1.5 (9)	C12—C13—C14—C15	0.0 (3)
C3—C4—C5—N1	179.4 (4)	C13—C14—C15—C16	−0.4 (3)
S1'—C4—C5—N1	177.1 (2)	C14—C15—C16—C17	0.3 (3)
S1—C4—C5—N1	0.8 (3)	C15—C16—C17—C12	0.2 (3)
C3'—C4—C5—O1	−179.8 (9)	C13—C12—C17—C16	−0.6 (3)
C3—C4—C5—O1	1.1 (4)	N4—C12—C17—C16	−178.96 (17)

### Hydrogen-bond geometry (Å, º)

Cg1 is the centroid of the O1,N1,N2,C5,C6 ring

**Table:**

*D*—H···*A*	*D*—H	H···*A*	*D*···*A*	*D*—H···*A*
C9—H9*B*···S2^i^	0.97	2.77	3.618 (3)	146
C1—H1···*Cg*1^ii^	0.93	2.72	3.545 (6)	148
C11—H11*A*···*Cg*1^iii^	0.97	2.98	3.600 (2)	123
C15—H15···*Cg*2^iii^	0.93	2.95	3.743 (3)	144

Symmetry codes: (i) −*x*+1/2, −*y*+3/2, −*z*+1; (ii) *x*+1/2, *y*+1/2, *z*; (iii) *x*+1/2, −*y*−1/2, *z*−1/2.
